# Supplementary material for: Immature olfactory sensory neurons provide behaviourally relevant sensory input to the olfactory bulb
Source: Nat Commun. 2022 Oct 19;13:6194. doi: 10.1038/s41467-022-33967-6 (PMC9582225; doi:10.1038/s41467-022-33967-6)
Supplement: Supplementary file 1 — Supplementary Information [file 41467_2022_33967_MOESM1_ESM.pdf]

Immature olfactory sensory neurons provide behaviourally relevant sensory input to the olfactory bulb

Jane S. Huang, Tenzin Kunkhyen, Alexander N. Rangel, Taryn R. Brechbill, Jordan D. Gregory, Emily D. Winson-Bushby, Beichen Liu, Jonathan T. Avon, Ryan J. Muggleton and Claire E.J. Cheetham

Supplementary Information

Supplementary Figures

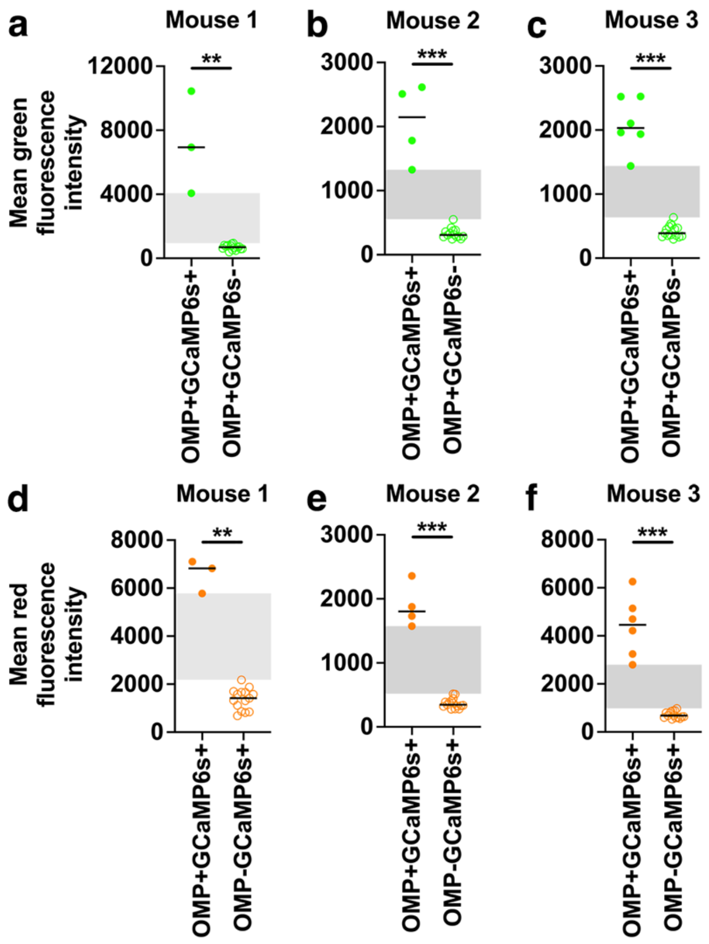

Figure S1. Fluorescence intensity of OSNs stained for Gγ8-GCaMP6s and/or OMP. **a – c** Intensity of green fluorescence (GFP staining to detect GCaMP6s expression) in OMP+GCaMP6s+ co-stained OSNs vs. OMP-expressing OSNs that did not express Gγ8-

GCaMP6s (OMP+GCaMP6s-) in OE sections from three mice. Lines: median, symbols: individual OSNs. Green fluorescence intensity was significantly different between OMP+GCaMP6s+ and OMP+GCaMP6s- cells in all three mice (Mann Whitney rank sum tests: **a** P = 0.003, U = 0, n = 3 vs. 15. **b** P < 0.001, U = 0, n = 4 vs. 15. **c** P < 0.001, U = 0 n = 6 vs. 15.) **d – f** Intensity of red fluorescence (OMP staining) in OMP+GCaMP6s+ co-stained OSNs vs. Gγ8-GCaMP6s-expressing OSNs that did not express OMP (OMP-GCaMP6s+) in OE sections from three mice. Lines: median, symbols: individual OSNs. Red fluorescence intensity was significantly different between OMP+GCaMP6+ and OMP-GCaMP6+ cells in all three mice (Mann Whitney rank sum tests: **d** P = 0.003, U = 0. **e** P < 0.001, U = 0. **f** P< 0.001, U = 0). n = 15 OMP+GCaMP6s- and 15 OMP-GCaMP6s+ OSNs per mouse; n = 3 (mouse 1), 4 (mouse 2) and 6 (mouse 3) OMP+GCaMP6s+ OSNs. Gray shaded regions indicate difference between the highest intensity cell in the non-co-expressing group and the lowest intensity cell in the co-expressing group. Differences in fluorescence intensity between mice are due to imaging at different times with different acquisition settings. All statistical tests were two-tailed. Source data are provided as a Source Data file.

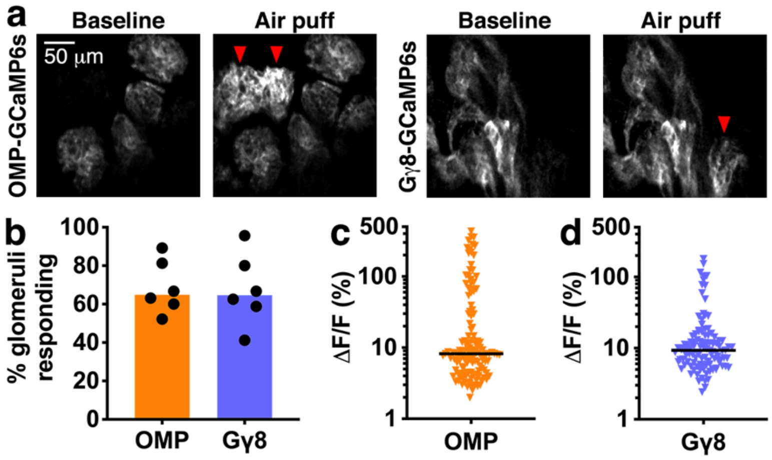

Figure S2. Immature and mature OSNs respond to a deodorized air puff stimulus.
a Example 2-photon images of single z-plane during single trial showing baseline and responses evoked by deodorized air puffs in OMP-GCaMP6s and Gγ8-GCaMP6s mice. Red arrows indicate air puff-responsive glomeruli. **b** Similar percentage of glomeruli

respond to air puff stimulus in OMP-GCaMP6s (median 64.9%) and Gγ8-GCaMP6s (median: 64.6%) mice (Mann-Whitney rank sum test. $P = 0.85$, $U = 16.5$, $n = 6$ mice per group). Bars: median, symbols: values for individual mice. **c** Amplitude of responses to air puff stimulation in OMP-GCaMP6s mice ($n = 131$ glomeruli from 6 mice). Line: median, symbols: values for individual glomeruli. **d** Amplitude of responses to air puff stimulation in Gγ8-GCaMP6s mice ($n = 107$ glomeruli from 6 mice). Line: median, symbols: values for individual glomeruli. All relevant statistical tests were two-tailed. Source data are provided as a Source Data file.

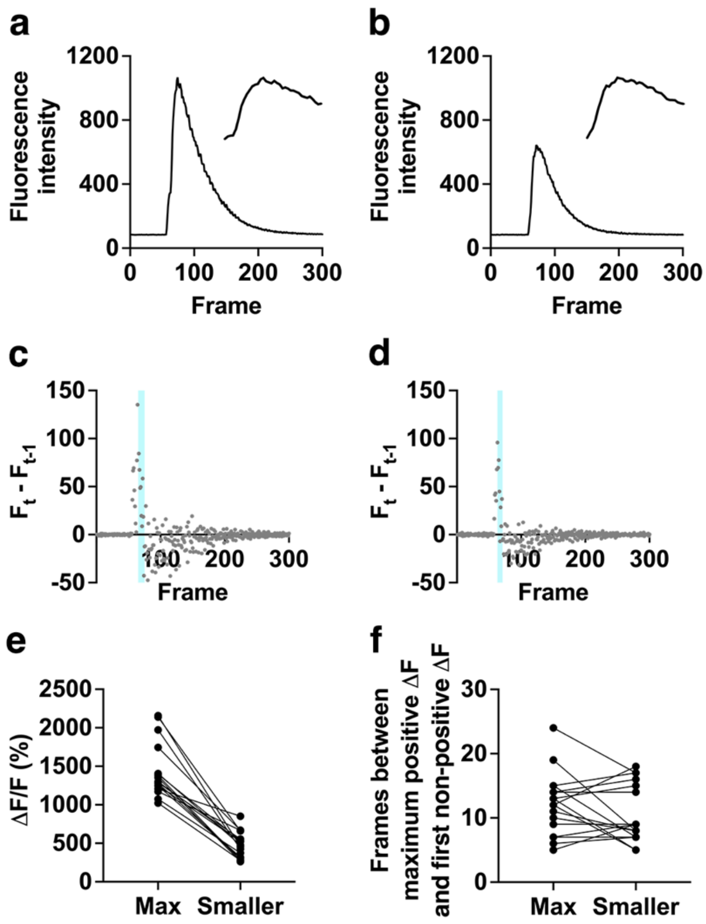

Figure S3. No evidence for GCaMP6s saturation in the largest odorant-evoked responses in OMP-GCaMP6s mice.

a Example of a maximal odorant-evoked response for a glomerulus from an OMP-GCaMP6s mouse (ΔF/F = 1173 %). **b** A smaller amplitude response (ΔF/F = 667 %) evoked by a lower concentration of the same odorant for the same glomerulus as shown in A. **a – b** Insets: zoomed traces of the response peak (frames 65-95) showing no plateau in the fluorescence intensity. **c** ΔF between frames for the maximal response shown in A. **d** ΔF between frames for the smaller response shown in B. **c – d** Shaded regions: time between the maximum positive ΔF and the first non-positive ΔF value. **e** Amplitudes of maximal vs. smaller responses in 17 analyzed glomeruli. **f** The time between the maximum positive ΔF and the first non-positive ΔF values is similar for maximal vs. smaller responses in the same glomeruli (Paired t-test. P = 0.20, t = 1.33, n = 17 glomeruli). None of the analyzed responses showed a very rapid transition from maximum

positive to non-positive ΔF values, indicating that GCaMP6s saturation had not occurred. All relevant statistical tests were two-tailed. Source data are provided as a Source Data file.

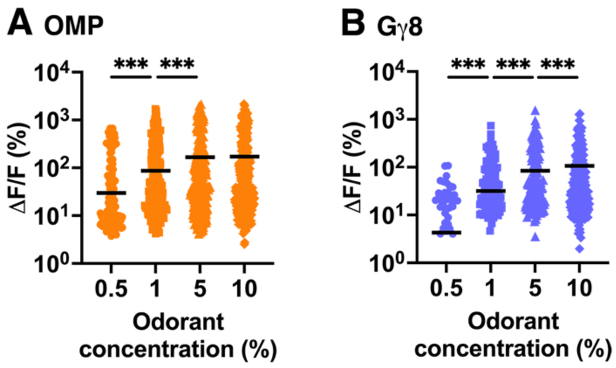

Figure S4. Analysis of glomerulus-odorant pairs shows that immature but not mature OSNs provide information about differences between odorant concentrations high in the tested range.

a Analysis of glomerulus-odorant pairs shows that odorant response amplitudes increase significantly between 0.5 % and 1 % concentrations, and 1 % and 5 % concentrations, but not between 5 % and 10 % concentrations in OMP-GCaMP6s mice (One-way repeated measures ANOVA. $P < 0.001$, $F_{1.59,611} = 81.1$. Sidak's multiple comparisons. 0.5 % vs. 1 %: $P < 0.001$, $t = 7.70$. 1 % vs. 5 %: $P < 0.001$, $t = 8.50$. 5 % vs. 10 %: $P = 0.49$, $t = 0.68$. n = 384 glomerulus-odorant pairs). **b** Analysis of glomerulus-odorant pairs shows that odorant response amplitudes increase significantly between each ascending pair of odorant concentrations in Gγ8-GCaMP6s mice (One-way repeated measures ANOVA. $P < 0.001$, $F_{1.32,299} = 58.7$. Sidak's multiple comparisons. 0.5 % vs. 1 %: $P < 0.001$, $t = 5.73$. 1 % vs. 5 %: $P < 0.001$, $t = 7.51$. 5 % vs. 10 %: $P < 0.001$, $t = 4.34$. n = 227 glomerulus-odorant pairs). **a – b** Lines: mean, symbols: glomerulus-odorant pairs. All relevant statistical tests were two-tailed. Source data are provided as a Source Data file.

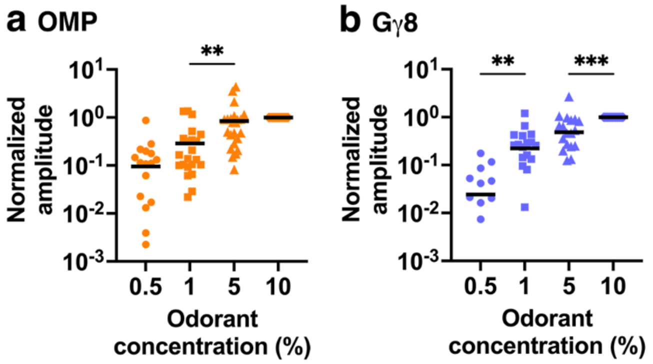

Figure S5. Analysis of normalized response amplitude shows that immature but not mature OSNs provide information about differences between odorant concentrations high in the tested range.

a Analysis of mouse-odorant pairs shows that normalized response amplitude increased significantly between 1 % and 5 % odorant concentrations in OMP-GCaMP6s mice (One-way repeated measures ANOVA. $P < 0.001$, $F_{1.11,\ 27.7} = 19.4$. Sidak's multiple comparisons. 0.5 % vs. 1 %: $P = 0.094$, $t = 2.26$. 1 % vs. 5 %: $P = 0.001$, $t = 4.07$. 5 % vs. 10 %: $P = 0.83$, $t = 0.78$. $n = 28$ mouse-odorant pairs). **b** Analysis of mouse-odorant pairs shows that normalized response amplitude increased significantly between 0.5 % and 1 % concentrations and between 5 % and 10 % concentrations in Gγ8-GCaMP6s mice (One-way repeated measures ANOVA. $P < 0.001$, $F_{1.39,\ 32.0} = 44.2$. Sidak's multiple comparisons. 0.5 % vs. 1 %: $P = 0.004$, $t = 3.70$. 1 % vs. 5 %: $P = 0.12$, $t = 2.14$. 5 % vs. 10 %: $P < 0.001$, $t = 4.36$. $n = 28$ mouse-odorant pairs). **a – b** Lines: mean, symbols: mouse-odorant pairs. All relevant statistical tests were two-tailed. Source data are provided as a Source Data file.

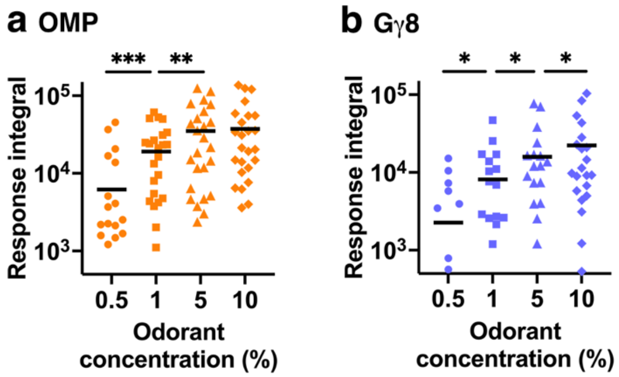

Figure S6. Analysis of response integral shows that immature but not mature OSNs provide information about differences between odorant concentrations high in the tested range.

a Analysis of mouse-odorant pairs shows that response integral increases significantly between 0.5 % and 1 % concentrations, and 1 % and 5 % concentrations, but not between 5 % and 10 % concentrations in OMP-GCaMP6s mice (One-way repeated measures ANOVA. $P < 0.001$, $F_{1.62,40.4} = 17.7$. Sidak's multiple comparisons. 0.5 % vs. 1 %: $P < 0.001$, $t = 5.45$. 1 % vs. 5 %: $P = 0.005$, $t = 3.55$. 5 % vs. 10 %: $P = 0.91$, $t = 0.59$. $n = 28$ mouse-odorant pairs. **b** Analysis of mouse-odorant pairs shows that response integral increases significantly between each ascending pair of odorant concentrations in Gγ8-GCaMP6s mice (One-way repeated measures ANOVA. $P = 0.002$, $F_{1.22,24.4} = 11.1$. Sidak's multiple comparisons. 0.5 % vs. 1 %: $P = 0.022$, $t = 2.98$. 1 % vs. 5 %: $P = 0.048$, $t = 2.62$. 5 % vs. 10 %: $P = 0.024$, $t = 2.95$. $n = 28$ mouse-odorant pairs. **a – b** Lines: mean, symbols: mouse-odorant pairs. All relevant statistical tests were two-tailed. Source data are provided as a Source Data file.

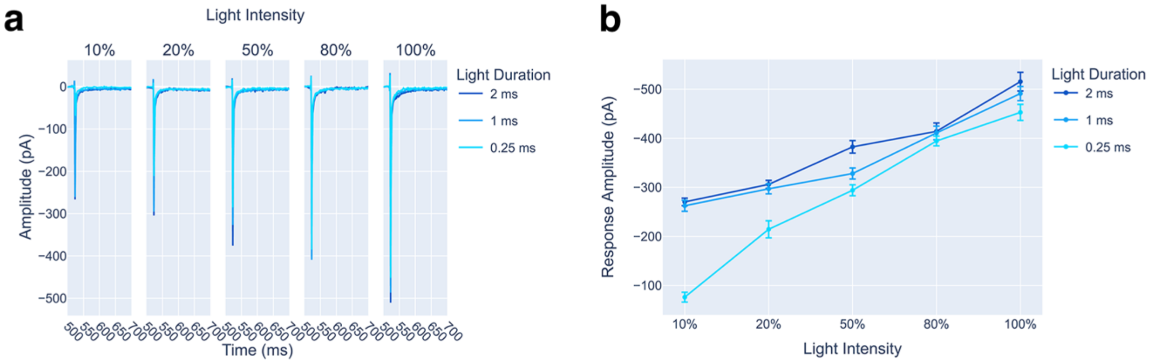

Figure S7. Power curve for STC responses to optogenetic stimulation in an OMP-ChIEF-Citrine mouse.

a Light-evoked EPSCs recorded in response to light pulses of increasing duration and light intensity in a single STC. Traces are averages of ten sweeps. **b** Relationship between light pulse intensity and duration and EPSC amplitude for the same STC. Symbols: mean, error bars: s.e.m. for 10 sweeps per stimulus condition.

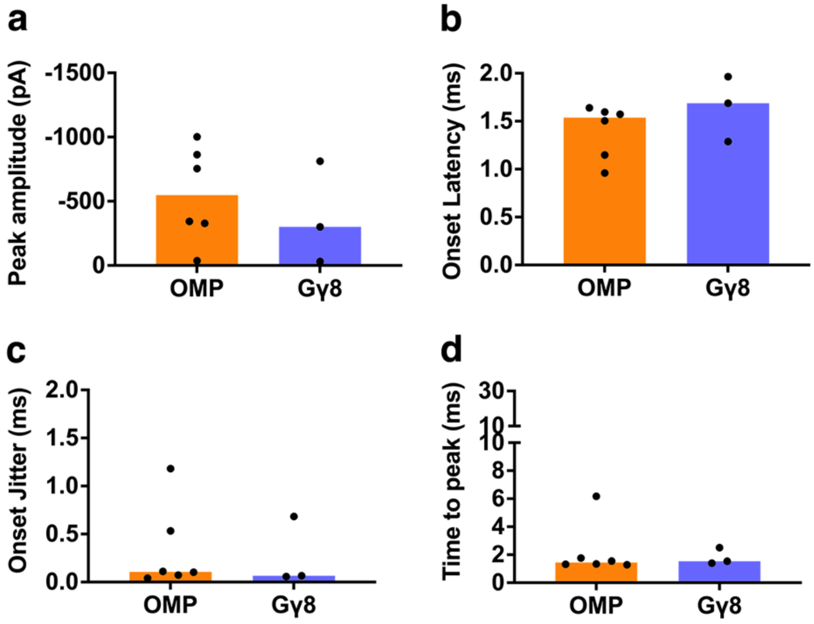

Figure S8. Properties of STC monosynaptic responses evoked by 1 ms duration, 50 % light intensity optogenetic stimulation of OMP-ChIEF-Citrine- and Gγ8-ChIEF-Citrine-expressing OSN axons

a Median peak amplitudes were not significantly different between OMP-ChIEF-Citrine and Gγ8-ChIEF-Citrine mice (Mann-Whitney test. $P = 0.38$, $U = 5$, $n = 6$ cells from 5 OMP-

ChIEF-Citrine mice, and n = 3 cells from 2 Gγ8-ChIEF-Citrine mice). Bars: median, symbols: values for individual neurons. **b** Median onset latency was not significantly different between OMP-ChIEF-Citrine and Gγ8-ChIEF-Citrine mice (Mann-Whitney test. P = 0.26, U = 4, n = 6 cells from 5 OMP-ChIEF-Citrine mice, and n = 3 cells from 2 Gγ8-ChIEF-Citrine mice). Bars: median, symbols: values for individual neurons. **c** Median onset jitter was not significantly different between OMP-ChIEF-Citrine and Gγ8-ChIEF-Citrine mice (Mann-Whitney test. P = 0.71, U = 7, n = 6 cells from 5 OMP-ChIEF-Citrine mice, and n = 3 cells from 2 Gγ8-ChIEF-Citrine mice). Bars: median, symbols: values for individual neurons. **d** Median time to peak was not significantly different between OMP-ChIEF-Citrine and Gγ8-ChIEF-Citrine mice (Mann-Whitney test. P = 0.71, U = 7, n = 6 cells from 5 OMP-ChIEF-Citrine mice, and n = 3 cells from 2 Gγ8-ChIEF-Citrine mice). Bars: median, symbols: values for individual neurons. Note that for the OMP-ChIEF-Citrine data set, one STC was present in Fig. 8 (1 ms, 100% intensity light stimulation) but lacked sweeps for 1 ms, 50% intensity light stimulation and so is not shown here. For the Gγ8-ChIEF-Citrine data set, two STCs were present both here and in Fig. 8, one STC present in Fig. 8 lacked sweeps for 1 ms, 50% intensity light stimulation and so is not shown here, and one STC from a different mouse lacked sweeps for 1 ms, 100% intensity light stimulation and so is present here but not in Fig. 8. All relevant statistical tests were two-tailed. Source data are provided as a Source Data file.

Supplementary Discussion

Role of putative mechanosensory input from immature OSNs

Recent studies suggest that mature OSNs can detect mechanical stimuli[1–5]. Our data from mature OSN axons expressing GCaMP6s were similar to a previous *in vivo* study that described airflow responses in tracheotomized mice[3], in terms of both the percentage of glomeruli that responded to the air puff and air puff response amplitude. The most parsimonious explanation for these data is therefore that our air puff stimulus, delivered to the external nares, also evokes mechanosensory responses. Importantly, we show that immature OSNs also exhibit putative mechanosensory responses similar to those in mature OSNs. This is not unexpected, in the context that immature OSNs respond to odorants and that mature OSNs employ the same OR-based signal transduction

machinery to generate both odorant and mechanosensory responses[2,4]. These data also suggest that responses to mechanical stimuli may be present as soon as immature OSNs begin to provide OB input, and hence that airflow-induced phase coding of odor identity[3] may also apply to sensory input received from immature OSNs. Another intriguing possibility is that mechanosensory-evoked activity could promote synapse formation and hence survival[6] of newborn OSNs. Most newborn OSNs do not survive beyond 14 days after terminal cell division in juvenile mice[7], perhaps because they must compete with both other immature OSNs and established mature OSNs to form synapses with postsynaptic targets. The role of odor-evoked activity in promoting the survival or integration of OSNs is unclear, as many of the ORs that are expressed will never encounter an odorant ligand[8]. In contrast, ORs are more likely to encounter mechanosensory stimuli, and a majority of glomeruli receive putative mechanosensory-evoked input (Fig. S2)[3].

Supplementary References

1. Carey, R. M., Verhagen, J. V., Wesson, D. W., Pírez, N. & Wachowiak, M. Temporal Structure of Receptor Neuron Input to the Olfactory Bulb Imaged in Behaving Rats. *J Neurophysiol* 101, 1073–1088 (2009).

2. Chen, X., Xia, Z. & Storm, D. R. Stimulation of Electro-Olfactogram Responses in the Main Olfactory Epithelia by Airflow Depends on the Type 3 Adenylyl Cyclase. *J Neurosci* 32, 15769–15778 (2012).

3. Iwata, R., Kiyonari, H. & Imai, T. Mechanosensory-Based Phase Coding of Odor Identity in the Olfactory Bulb. *Neuron* 96, 1139–1152 (2017).

4. Connelly, T. *et al.* G protein-coupled odorant receptors underlie mechanosensitivity in mammalian olfactory sensory neurons. *Proc Natl Acad Sci* 112, 590–595 (2015).

5. Grosmaitre, X., Santarelli, L. C., Tan, J., Luo, M. & Ma, M. Dual functions of mammalian olfactory sensory neurons as odor detectors and mechanical sensors. *Nature Neuroscience* 10, 348–354 (2007).

6. Schwob, J. E., Szumowski, K. E. & Stasky, A. A. Olfactory sensory neurons are trophically dependent on the olfactory bulb for their prolonged survival. *J Neurosci* 12, 3896–3919 (1992).

7. Savya, S. P., Kunkhyen, T. & Cheetham, C. Low survival rate of young adult-born olfactory sensory neurons in the undamaged mouse olfactory epithelium. *J. Bioenerg. Biomembr.* 51, 41–51 (2019).

8. Saraiva, L. R. *et al.* Hierarchical deconstruction of mouse olfactory sensory neurons: from whole mucosa to single-cell RNA-seq. *Scientific reports* 5, 18178 (2015).
