# Supplementary material for: Immature olfactory sensory neurons provide behaviourally relevant sensory input to the olfactory bulb
Source: Nat Commun. 2022 Oct 19;13:6194. doi: 10.1038/s41467-022-33967-6 (PMC9582225; doi:10.1038/s41467-022-33967-6)
Supplement: Supplementary file 3 — Description of Additional Supplementary Files [file 41467_2022_33967_MOESM3_ESM.docx]

**Description of Additional Supplementary Files**

**Supplementary Dataset 1. Key Resources.**

Includes details of antibodies, chemicals, mouse strains, oligonucleotides and software used.
