# Supplementary material for: Immature olfactory sensory neurons provide behaviourally relevant sensory input to the olfactory bulb
Source: Nat Commun. 2022 Oct 19;13:6194. doi: 10.1038/s41467-022-33967-6 (PMC9582225; doi:10.1038/s41467-022-33967-6)
Supplement: Supplementary file 5 — Reporting Summary [file 41467_2022_33967_MOESM5_ESM.pdf]

nature research

Corresponding author(s): Claire Cheetham

Last updated by author(s): Sep 21, 2022

Reporting Summary

Nature Research wishes to improve the reproducibility of the work that we publish. This form provides structure for consistency and transparency in reporting. For further information on Nature Research policies, see our Editorial Policies and the Editorial Policy Checklist.

Statistics

For all statistical analyses, confirm that the following items are present in the figure legend, table legend, main text, or Methods section.

n/a	Confirmed	
☐	☒	The exact sample size (*n*) for each experimental group/condition, given as a discrete number and unit of measurement
☐	☒	A statement on whether measurements were taken from distinct samples or whether the same sample was measured repeatedly
☐	☒	The statistical test(s) used AND whether they are one- or two-sided<br>*Only common tests should be described solely by name; describe more complex techniques in the Methods section.*
☐	☒	A description of all covariates tested
☐	☒	A description of any assumptions or corrections, such as tests of normality and adjustment for multiple comparisons
☐	☒	A full description of the statistical parameters including central tendency (e.g. means) or other basic estimates (e.g. regression coefficient) AND variation (e.g. standard deviation) or associated estimates of uncertainty (e.g. confidence intervals)
☐	☒	For null hypothesis testing, the test statistic (e.g. *F*, *t*, *r*) with confidence intervals, effect sizes, degrees of freedom and *P* value noted<br>*Give P values as exact values whenever suitable.*
☒	☐	For Bayesian analysis, information on the choice of priors and Markov chain Monte Carlo settings
☒	☐	For hierarchical and complex designs, identification of the appropriate level for tests and full reporting of outcomes
☒	☐	Estimates of effect sizes (e.g. Cohen's *d*, Pearson's *r*), indicating how they were calculated

Our web collection on statistics for biologists contains articles on many of the points above.

Software and code

Policy information about availability of computer code

Data collection	Python (Pyzo 4.4.2), Nikon NIS-Elements 3.10, ThorImage 4.0 and ThorSync 4.0, SlideBook 6, Igor Pro 9, Echo 6.4.1, Olympus FV 10ASW
Data analysis	GraphPad Prism 8 and 9, Fiji v. 1.48. Analysis code is available at: https://doi.org/10.5281/zenodo.7013674 (image acquisition and analysis) and https://doi.org/10.5281/zenodo.6993495 (electrophysiology analysis)

For manuscripts utilizing custom algorithms or software that are central to the research but not yet described in published literature, software must be made available to editors and reviewers. We strongly encourage code deposition in a community repository (e.g. GitHub). See the Nature Research guidelines for submitting code & software for further information.

Data

Policy information about availability of data

All manuscripts must include a data availability statement. This statement should provide the following information, where applicable:

- Accession codes, unique identifiers, or web links for publicly available datasets
- A list of figures that have associated raw data
- A description of any restrictions on data availability

The data that support the findings of this study are available in Zenodo with the identifier https://doi.org/10.5281/zenodo.7154187. Source data for relevant figure panels in Fig. 1-7, Fig. S1-S4 and Fig. S5 are provided with this paper.

Field-specific reporting

Please select the one below that is the best fit for your research. If you are not sure, read the appropriate sections before making your selection.

☒ Life sciences ☐ Behavioural & social sciences ☐ Ecological, evolutionary & environmental sciences

For a reference copy of the document with all sections, see nature.com/documents/nr-reporting-summary-flat.pdf

Life sciences study design

All studies must disclose on these points even when the disclosure is negative.

Sample size	Power analyses using power = 80%, alpha = 0.05, a 25% effect size (ES), and standard deviation = ES/2, indicated a minimum of 3 mice per experimental group. For many experiments, our sample size exceeded this minimum value.
Data exclusions	No data were excluded from the analyses
Replication	All attempts at replication were successful. Behavior experiments were performed on three separate cohorts of mice and data were pooled. The number of animals from which data were derived is stated for each data set (see figure legends).
Randomization	Mice were randomly allocated to treatment groups.
Blinding	Researchers were blinded to group allocation where possible. For electrophysiology and in vivo 2-photon imaging experiments this was not feasible due to the small number of personnel in the lab, including due to Covid-19 mitigation measures, and because the genotype (OMP-GCaMP6s vs. Gg8-GCaMP6s) was apparent from visualization of axons necessary to perform the experiments. Data analysis was performed blinded to group allocation and/or results were confirmed by an additional investigator who was blinded to group allocation.

Reporting for specific materials, systems and methods

We require information from authors about some types of materials, experimental systems and methods used in many studies. Here, indicate whether each material, system or method listed is relevant to your study. If you are not sure if a list item applies to your research, read the appropriate section before selecting a response.

Materials & experimental systems

n/a	Involved in the study	
☐	☒	Antibodies
☒	☐	Eukaryotic cell lines
☒	☐	Palaeontology and archaeology
☐	☒	Animals and other organisms
☒	☐	Human research participants
☒	☐	Clinical data
☒	☐	Dual use research of concern

Methods

n/a	Involved in the study	
☒	☐	ChIP-seq
☒	☐	Flow cytometry
☒	☐	MRI-based neuroimaging

Antibodies

Antibodies used	Goat polyclonal anti-olfactory marker protein Wako Chemicals #544-10001<br>RRID:AB_664696<br>Rabbit polyclonal anti-GAP43 Novus Biologicals NB300-143<br>RRID:AB_10001196<br>Donkey anti-goat IgG (H+L) Alexa Fluor 546 conjugated Thermo Fisher Scientific A11056<br>RRID:AB_142628<br>Donkey anti-rabbit IgG (H+L) Alexa Fluor 546 conjugated Thermo Fisher Scientific A10040<br>RRID:AB_2534016<br>Donkey anti-rabbit IgG (H+L) Alexa Fluor 647 conjugated Thermo Fisher Scientific A31573<br>RRID:AB_2536183<br>GFP-Booster-Atto-488 Chromotek gba-488-100<br>RRID:AB_2631386
Validation	Anti-olfactory marker protein: validation provided on manufacturer's website, which includes relevant references: https://labchem-wako.fujifilm.com/us/product_data/docs/US00000001_doc01.pdf.<br>Anti-GAP43: manufacturer provides validation data on their website along with relevant publications: https://www.novusbio.com/products/gap-43-antibody_nb300-143<br>GFP-Booster: specificity data available on manufacturer's website: https://www.chromotek.com/fileadmin/content/PDFs/Fluorescent_Protein_Specificity_Table/Fluorescent_protein_specificity_table_Nano-Booster_200109.pdf

Animals and other organisms

Policy information about studies involving animals; ARRIVE guidelines recommended for reporting animal research

Laboratory animals	C57BL/6J mice (strain #000664) were purchased from the Jackson Laboratory and all other lines were bred in-house. Mice were maintained on a 12 h light/dark cycle in individually ventilated cages at 22C and 48 % humidity with unrestricted access to food and water unless otherwise stated. Mice were group-housed if same sex littermates were available.<br>Generation of the G8-tTA (Nguyen et al., 2007), tetO-sypGFP-tdT (Li et al., 2010), OMP-IRES-tTA (Yu et al., 2004), tetO-ChIEF-Citrine (Cheetham et al., 2016), tetO-GCaMP6s (Wekselblatt et al., 2016), OMP-Cre (Li et al., 2004), Ai9 (Madisen et al., 2009) and M72-RFP Zhang et al., 2012) mouse lines has been described previously. Mice for 2-photon calcium imaging experiments were G8-GCaMP6s [G8-tTA+/-;tetO-GCaMP6s+/-], OMP-GCaMP6s [OMP-tTA+/-;tetO-GCaMP6s+/-], G8-GCaMP6s-OMP-tdT [G8-tTA +/-;tetO-GCaMP6s+/-;OMP-cre+/-;flox-tdT+/-], OMP- GCaMP6s-OMP-tdT [OMP-tTA+/-;tetO-GCaMP6s+/-;OMP-cre+/-;flox-tdT+/-], G8-GCaMP6s-M72-RFP [G8-tTA+/-;tetO-GCaMP6s+/-;M72-IRES-RFP+/+] or OMP-GCaMP6s-M72-RFP [OMP-tTA+/-;tetO-GCaMP6s+/-;M72-IRES-RFP+/+]. Mice for electrophysiology experiments were G8-ChIEF-Citrine [G8-tTA+/-;tetO-ChIEF-Citrine +/-] or OMP-ChIEF-Citrine [OMP-tTA+/-;tetO-ChIEF-Citrine+/-]. Mice for behavioral experiments were C57BL/6J (The Jackson Laboratory). All genetically modified mice were of mixed 129 x C57BL/6J background, and each experimental group comprised approximately equal numbers of male and female mice, which were randomly assigned to experimental groups. P21-23 mice were used for 2-photon imaging and associated histology. P18-25 mice were used for slice electrophysiology and associated histology. 8-week-old mice were used for behavior experiments and retrograde tracer injections.
Wild animals	No wild animals were used in the study
Field-collected samples	No field-collected samples were used in the study
Ethics oversight	All animal procedures conformed to National Institutes of Health guidelines and were approved by the Carnegie Mellon University and University of Pittsburgh Institutional Animal Care and Use Committees.

Note that full information on the approval of the study protocol must also be provided in the manuscript.
